# Prolonged *ex vivo* lung perfusion with corrected perfusate: functional and structural benefits in swine

**DOI:** 10.3389/frtra.2026.1899960

**Published:** 2026-08-19

**Authors:** Justin Issard, Julia Mercier, Jean-Baptiste Ménager, Guillaume Fadel, Yuya Nobori, Melwan Izem, Maria-Rosa Ghigna, Elie Fadel, Fabrice Antigny, Pierre Mordant, Olaf Mercier

**Affiliations:** 1 Department of Thoracic Surgery and Heart-Lung Transplantation, Marie Lannelongue Hospital, Université Paris-Saclay, Le Plessis Robinson, France; 2 Hypertension Pulmonaire: Physiopathologie et Innovation Thérapeutique (HPPIT), Hôpital Bicêtre (AP-HP), Hôpital Marie Lannelongue, FHU André Cournand, ERN-LUNG, Université Paris-Saclay, INSERM UMR_S 1358, Le Plessis Robinson, Le Kremlin-Bicêtre, France; 3 Department of Pathology, Marie Lannelongue Hospital, Groupe Hospitalier Paris-Saint-Joseph, Le Plessis Robinson, France; 4 Department of Thoracic and Vascular Surgery and Lung Transplantation, Bichat Hospital, Assistance-Publique des Hôpitaux de Paris, Paris, France

**Keywords:** EVLP, EVLP duration, lung transplant (LTx), pig model, preclinical (*in vivo*) studies, prolonged EVLP

## Abstract

**Background:**

During *ex vivo* lung perfusion (EVLP), perfusate pH, electrolyte, and glucose concentrations often deviate from physiological ranges. We evaluated the effect of a corrective solution on maintaining these parameters within physiological limits during 12 h of EVLP in a porcine model.

**Methods:**

Twelve porcine lung blocks were harvested and cold-preserved before undergoing 12 h of normothermic EVLP. Lung blocks were randomly assigned to two groups based on the perfusate replacement strategy. All lungs were perfused with Steen solution, mean priming volume was 1,525 ± 340 and 1,325 ± 232 mL, with 100 mL (5%–9%) replaced every two hours by either fresh Steen solution (standard group, *n* = 6) or a modified solution designed to restore physiological composition (corrected group, *n* = 6). Perfusate electrolytes, glucose, cytokines, pH, partial oxygen pressure (PO₂), pulmonary vascular resistance (PVR), and compliance were measured. After EVLP, left lungs were transplanted during 4 h into donor-related pigs, while right lungs were analyzed by histology and electron microscopy.

**Results:**

Perfusate correction successfully maintained electrolyte, pH, and glucose levels within target ranges during EVLP. The corrected group demonstrated higher lung compliance and oxygenation, reduced edema, more hyaline membranes, and a greater density of Kohn's pores after EVLP. PVR was similar between groups, and no significant difference in *Δ*PO₂ was observed post-transplantation.

**Conclusion:**

Maintaining physiological perfusate composition improved lung function during 12-hour EVLP in swine; however, no post-transplant benefit was observed. Further studies are warranted to assess the impact of this strategy during prolonged EVLP and to compare it with alternative approaches.

## Graphical Abstract

**Figure:**
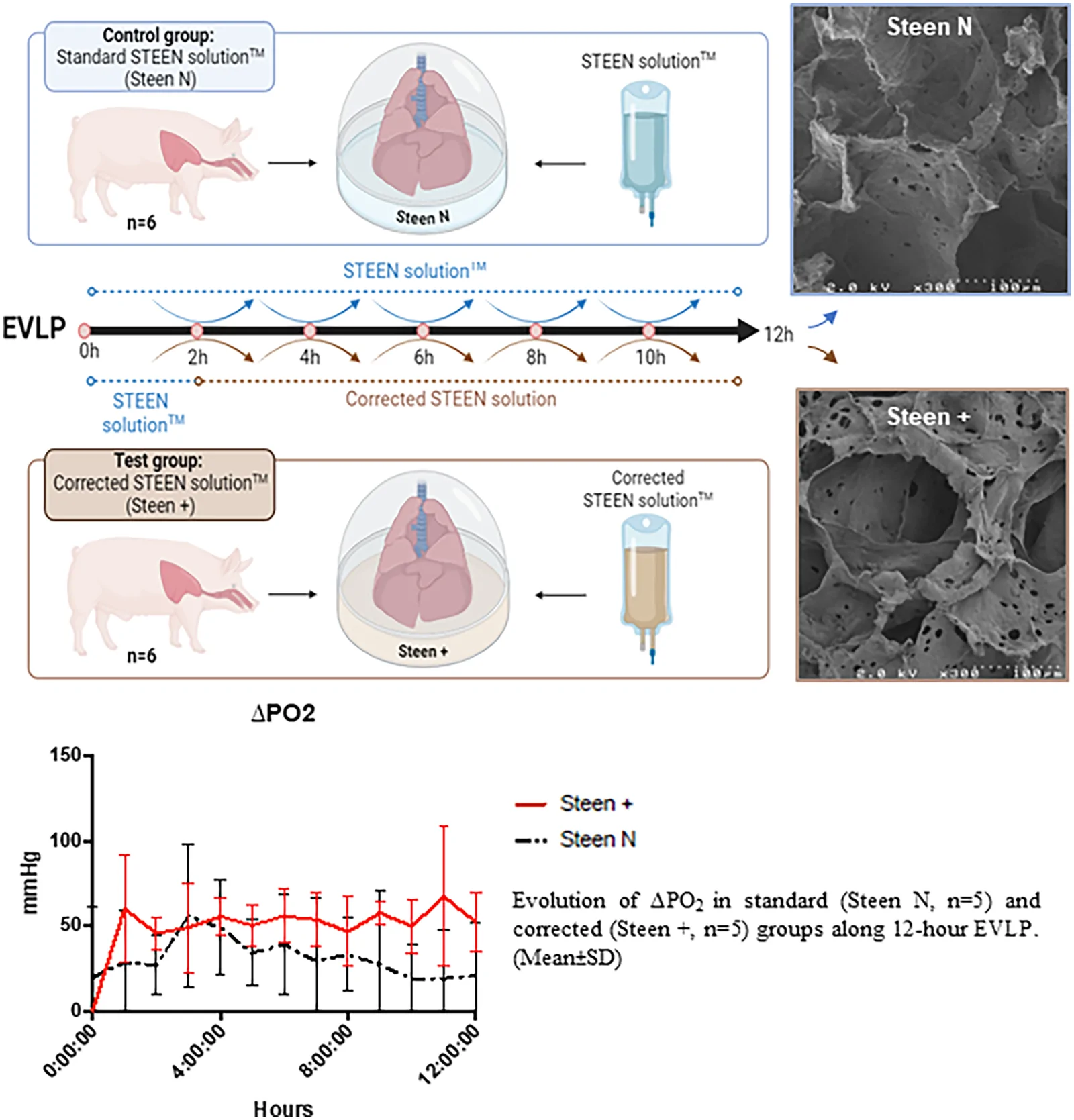
Created in BioRender. Mercier, J. (2026) https://BioRender.com/c6gjmtr.

## Highlights

- In swine, perfusate electrolytes, pH, and glucose were corrected during EVLP.
- This correction over a 12-hour period was feasible and safe.
- Lung compliance and oxygenation were better with vs. without correction.
- Outcomes of transplantation after EVLP were similar with vs. without correction.

## 1 Introduction

The shortage of lung grafts has led to the widespread acceptance of marginal grafts from donors after circulatory death (DCDD) for transplantation (1). Although this approach increases graft availability, these lungs are susceptible to hypoxia-related injury and require functional assessment prior to transplantation. Normothermic *ex vivo* lung perfusion (EVLP) enables evaluation of high-risk lungs and, when applied to DCDD lungs, has achieved outcomes comparable to those from donors after neurological determination of death (DNDD) (2–5). EVLP appears to improve lung oxygenation (3, 6) and clinical outcomes, including reduced severity of primary graft dysfunction (5), shorter mechanical ventilation, and decreased hospital stay compared with static cold storage (7). Extending EVLP duration could allow lung reconditioning interventions such as antibiotic therapy, gene transfer via adenoviral vectors, immunomodulation, or mesenchymal stem cell administration (8–11), making it a major research focus (12, 13). However, EVLP has been associated with inflammatory responses and cellular injury (14) and is typically limited to 4–6 h in clinical practice (15, 16). Progressive deterioration of lung function remains the main barrier to prolonged EVLP (8, 12, 17). Interestingly, intermittent EVLP strategies, in which normothermic perfusion is alternated with periods of cold preservation, have emerged as a promising approach to extend total preservation time while limiting continuous perfusion-related injury (18). Under these conditions, acellular perfusate has been linked to stable lung function and favorable post-transplant outcomes in both swine and humans (19). Such strategies may reduce cumulative endothelial stress, metabolic exhaustion, and edema formation compared with uninterrupted EVLP. The longest successful EVLP reported in a porcine model achieved lung preservation for up to three days (20). Currently, EVLP is most commonly performed using the Toronto protocol (5), which employs Steen solution (Perfadex®, XVIVO, Gothenburg, Sweden) as the perfusate. Steen solution contains electrolytes at extracellular concentrations, human albumin for oncotic pressure, and dextran 40 for endothelial protection (21). It can be mixed with donor blood to create a cellular perfusate with a hematocrit of 15%, which has been evaluated for prolonged EVLP (12, 22). Whole donor blood has maintained stable lung function for 24-hour EVLP in swine (12, 18), although cellular perfusate has been associated with increased pulmonary vascular resistance (PVR) during EVLP (7). Other perfusion solutions, such as OCS™ solution (TransMedics, Andover, MA), are also available (12). Despite these advances, EVLP duration in humans remains limited. One strategy to extend EVLP is to optimize the perfusate composition (13). During prolonged EVLP, electrolyte concentrations deviate from physiological ranges (23), pH decreases due to lactate accumulation and membrane-induced deoxygenation, and glucose depletion is associated with edema and impaired cellular function (24). To counter these changes, the Toronto protocol replaces 100 mL of Steen solution with fresh solution every two hours during 12-hour EVLP (19). We hypothesized that replacing Steen solution with a corrective solution designed to maintain electrolyte, pH, and glucose levels within physiological ranges would be more effective. Therefore, we conducted a porcine study to determine whether such a solution improves functional and structural lung parameters during 12-hour EVLP.

## 2 Materials and methods

### 2.1 Study design

**Figure 1:**
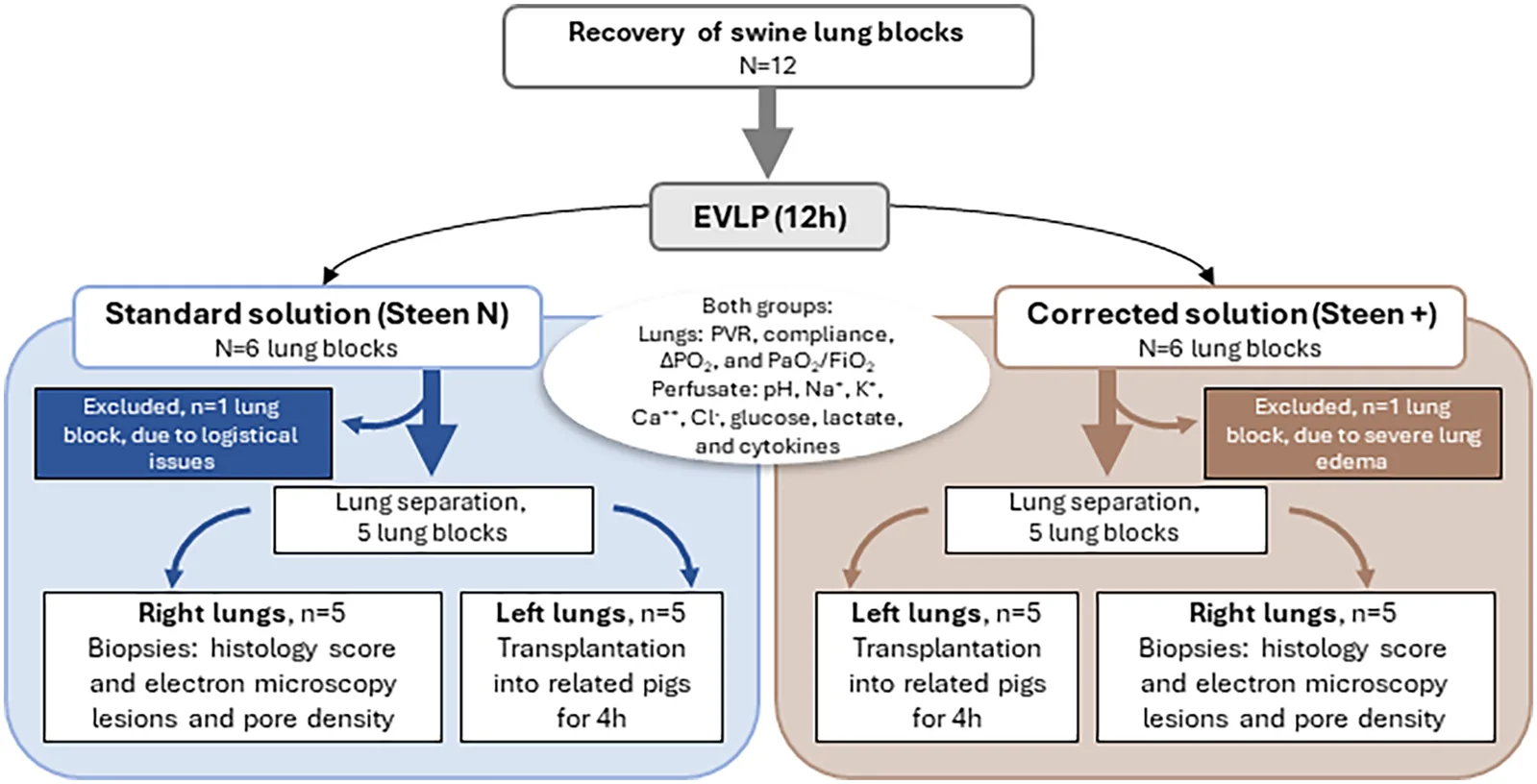
Study design of 12-hours EVLP in a porcine model, comparing standard steen solution with a corrected perfusate. 12 swine lung blocks were recovered and allocated equally into two groups. Both groups were perfused with Steen solution the first two hours_:_ then perfusate was exchanged every 2 h. Standard group: 100 mL of perfusate was exchanged for 100 mL of Steen solution_:_ according to the Toronto protocol Corrected group: 100 mL of perfusate was replaced by 100 mL of a solution composed of one-third Steen solution, one-third 2.5% glucose solution_:_ and one-third sodium bicarbonate solution; all other components of EVLP were according to the Toronto protocol.

The study design is shown in Figure 1. Twelve lung blocks from donor pigs were harvested and subjected to 12-hour EVLP according to the Toronto protocol. Six lungs were perfused with Steen solution (standard group), and six with a corrective solution formulated to maintain pH, electrolytes, and glucose within physiological ranges (corrected group). At the end of EVLP, right lungs were analyzed by optical and electron microscopy. Ten left lungs were deemed suitable for transplantation into donor-related pigs. In total, 22 female piglets (32–55 kg) were included. All animals received humane care in compliance with French and European regulations. Animal experiments were approved by the institutional ethics committee (C2EA-26/SBEA; APAFiS #2016111716212529) and conducted in full compliance with European Directive 2010/63/EU on the protection of animals used for scientific purposes.

### 2.2 Lung procurement and perfusion technique

Animals underwent general anesthesia, and the heart–lung block was retrieved and stored in a sterile organ bag at 4 °C filled with Steen solution until EVLP initiation (Table 1). EVLP was performed following the Toronto EVLP protocol (Cypel et al., J Heart Lung Transplant 2008) (19), with the only difference being the perfusate replacement strategy. In the standard group, 100 mL of Steen solution (5%–10% of total priming volume; Table 1) was exchanged every 2 h. In the corrected group, 100 mL of a mixture consisting of one-third Steen solution, one-third 2.5% glucose solution (Glucose B Braun 2.5%, 250 mL), and one-third sodium bicarbonate solution (14 mg/mL; Bicarbonate de Sodium Baxter 1.4%, 0.14 g/10 mL) was added every 2 h to maintain physiological levels of sodium, potassium, calcium, chloride, pH, and glucose (25). The corrective solution was designed to address electrolyte and metabolic imbalances, specifically abnormalities in potassium (K⁺), chloride (Cl⁻), and glucose levels, while maintaining physiological pH and oncotic pressure. Target values were defined as follows: pH 7.3–7.5, potassium 3.8–4.2 mmol/L, chloride <120 mmol/L, and glucose >10 mmol/L. After 12 h of EVLP, lungs were cooled to 10 °C and stored at 4 °C until transplantation. First and second cold ischemia times are reported in Table 1.

**Table 1:** Features of the animals, grafts, and graft preservation in the groups with vs. without perfusate composition correction.

Variables, mean ± SD	Standard^a^ *n* = 5	Correction^b^ *n* = 5	*P*-value
Lung recovery			
Weight of the donor animal, kg	38 ± 5	41 ± 9	0.57
Warm ischemia time, min	23 ± 6	20 ± 5	0.56
Anterograde pneumoplegia, mL	1,667 ± 289	1,475 ± 61	0.37
Retrograde pneumoplegia, mL	600 ± 173	542 ± 102	0.63
Cold ischemia time before EVLP, min	89 ± 20	81 ± 20	0.59
Lung weight, g	483 ± 126	557 ± 151	0.47
EVLP			
Priming volume, mL	1,525 ± 340	1,325 ± 232	0.35
Perfusate volume replaced during EVLP, mL	725 ± 154	790 ± 152	0.83
EVLP duration, min	695 ± 57	694 ± 30	0.81
Lung weight at the end of EVLP, g	883 ± 293	835 ± 287	0.82
Cold ischemia time after EVLP, min	331 ± 63	352 ± 64	0.68

EVLP, ex-vivo lung perfusion.

a Steen solution with exchange of 100 mL every 2 h.

b Steen solution with replacement of 100 mL every 2 h by a solution composed of one-third Steen solution, one-third 2.5% glucose solution, and one-third sodium bicarbonate (1.4%) solution.

### 2.3 Data collection during EVLP

Variables recorded before, hourly during, and after EVLP included:

- Functional parameters: pulmonary vascular resistance [PVR=(mPAP – LAP)/CO], dynamic compliance (ventilator-derived), *Δ*PO₂ between pulmonary artery (PA) and left atrium (LA), and PaO₂/FiO₂ ratio.
- Perfusate composition: pH, glucose, lactate, Na⁺, K⁺, Cl⁻, and Ca^2^⁺ concentrations.

Perfusate samples were collected from the LA, except for pH and blood gas analysis, which were performed in both PA and LA. Lungs were weighed before and after EVLP. Cytokine levels (IL-6, IL-18, TNF-α, IL-1β, IL-10, IL-1*α*, IL-2, IL-8, IL-12) were measured every 2 h using the Milliplex MAP Porcine Cytokine/Chemokine Magnetic Bead Panel (Merck Millipore, Burlington, MA) according to manufacturer instructions. Results are expressed in pg/mL.

### 2.4 Electron microscopy, histology, elastometry, and endothelial & mitochondrial protein expression

After EVLP, biopsies were taken from two lobes of the right lung (upper/middle and lower). Upper or middle lobe samples were used for electron microscopy to minimize edema. Samples were inflated with 2% glutathione and 0.1 M sodium cacodylate, stored at 4 °C for 48 h, then processed for transmission and scanning electron microscopy. Images were analyzed using ImageJ (https://imagej.net/ij/). The number of alveolar pores (pores of Kohn) was quantified on scanning electron microscopy slides. Lower lobe samples were fixed in 10% buffered formalin, embedded in paraffin, sectioned (5 µm), and stained with hematoxylin and eosin. A semi-quantitative histological score (0–12) was applied for interstitial edema, alveolar edema, cellular infiltration, and hyaline membrane formation (12, 19, 20). Histological analysis was performed on five sections from three animals per group due to tissue availability constraints at the time of analysis (samples from two animals were consumed by electron microscopy preparation). Sample selection was not randomized but was based on tissue quality and availability. Pulmonary pathologist (MG) was blinded to group allocation during scoring. Pulmonary artery elastometry was performed on the first branch of the right lung after EVLP and compared with control arteries from the explanted left lung. Contractility and relaxation were assessed using thromboxane A₂ mimetic, KCl, sildenafil, and acetylcholine. Western blot analyses were performed on tissue samples from control lungs and from lungs perfused for 12 h with either standard Steen solution (Steen N, *n* = 5) or corrected Steen solution (Steen+, *n* = 5). Membranes were incubated overnight at 4 °C with primary antibodies against phosphorylated endothelial nitric oxide synthase (phospho-eNOS), endothelial nitric oxide synthase (NOS3), Twist, vascular endothelial cadherin (VE-cadherin), mitochondrial transcription termination factor (MTERF), and cytochrome c oxidase subunit 15 (COX15). *β*-actin was used as the loading control. Immunoreactive bands were visualized using enhanced chemiluminescence and imaged using a digital detection system. Band intensities were quantified by densitometric analysis and normalized to *β*-actin expression.

### 2.5 Single-lung transplantation

In recipients, a left thoracotomy was performed in the fourth intercostal space, and the left lung was removed. Donor lungs were separated after pulmonary hilum dissection and ligation of the left azygos vein. Anastomoses were performed using running polypropylene sutures (Prolene, Ethicon Inc., Raritan, NJ): 4-0 for bronchus, 5-0 for LA, and 6-0 for PA. The graft was de-aired through the LA anastomosis. Recipients were ventilated through a single endotracheal tube without selective intubation. Post-transplant respiratory parameters (PVR, compliance, *Δ*PO₂, PaO₂/FiO₂) were compared between groups. To obtain graft-specific data blood samples were collected from the left pulmonary artery and the upper left pulmonary vein intraoperatively.

### 2.6 Statistical analysis

Statistical analyses were performed using Prism 9 (GraphPad Software, La Jolla, CA). Quantitative variables are expressed as median ± SD and compared using Student's *t*-test. Categorical variables are expressed as *n* (%) and compared using chi-square or Fisher's exact test. Time-course data were analyzed using a two-way repeated-measures ANOVA with group and time as factors, allowing for the assessment of longitudinal changes while accounting for within-animal repeated measurements. A *p*-value < 0.05 was considered statistically significant.

## 3 Results

### 3.1 Lung procurement and EVLP

Of the 12 heart–lung blocks, six were perfused using the standard Toronto protocol (100 mL exchanges every 2 h) and six using the corrected perfusate strategy (100 mL replacement every 2 h). Data from two animals (one per group) were excluded due to severe lung edema and logistical issues. Consequently, ten pigs underwent left-lung transplantation after 12-hour EVLP (Figure 1). The volume of perfusate exchanged during EVLP did not differ significantly between groups (*P* = 0.14). Baseline characteristics of animals, grafts, and EVLP procedures were comparable between groups (Table 1).

### 3.2 Physiological and biochemical findings during EVLP

**Figure 2:**
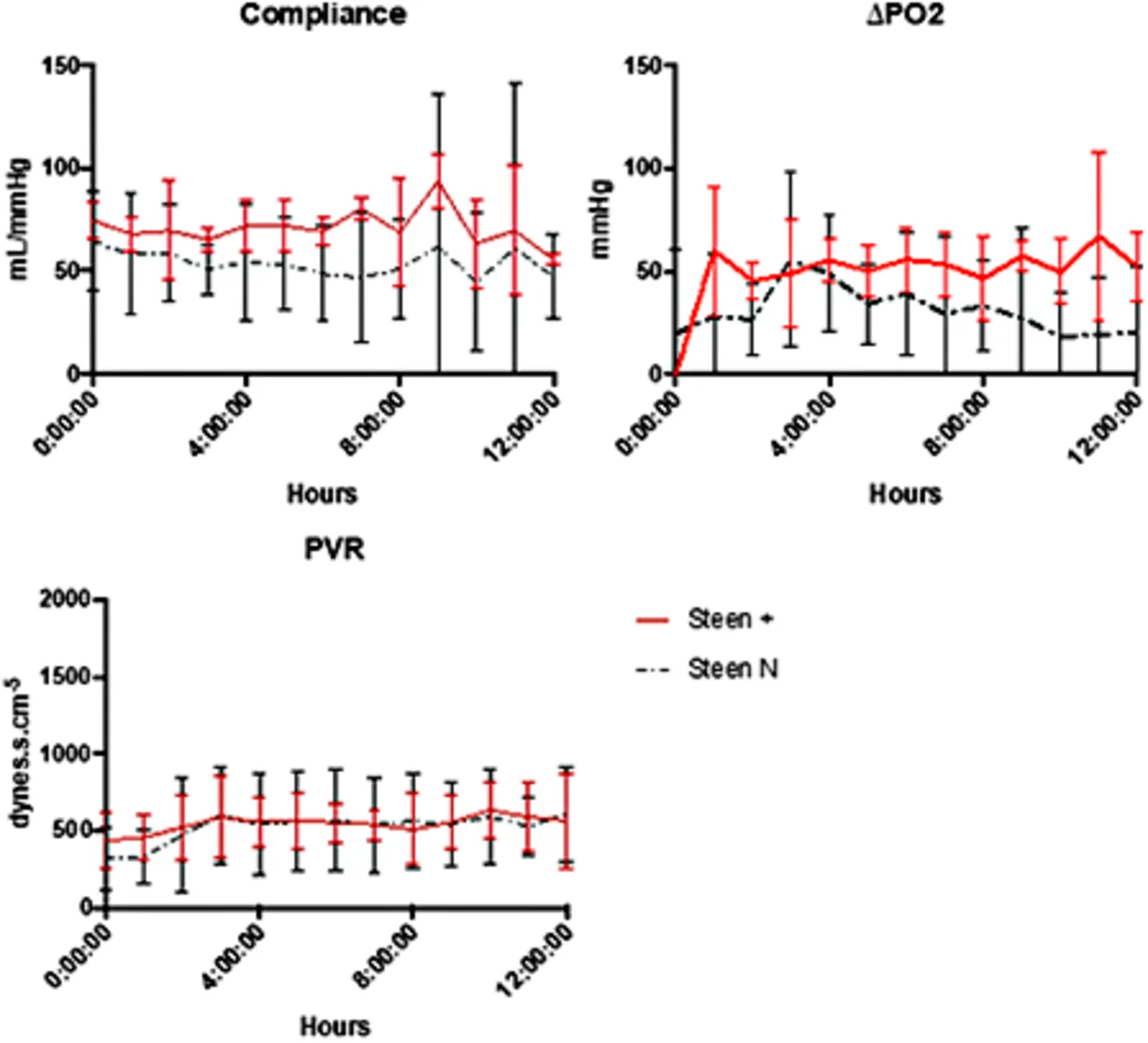
Evolution of compliance, APOL2 and FIR in standard (steen N, *n*=5) and corrected (steen *n*=5) groups along 12-hour EVIP (Mean ± SD).

**Figure 3:**
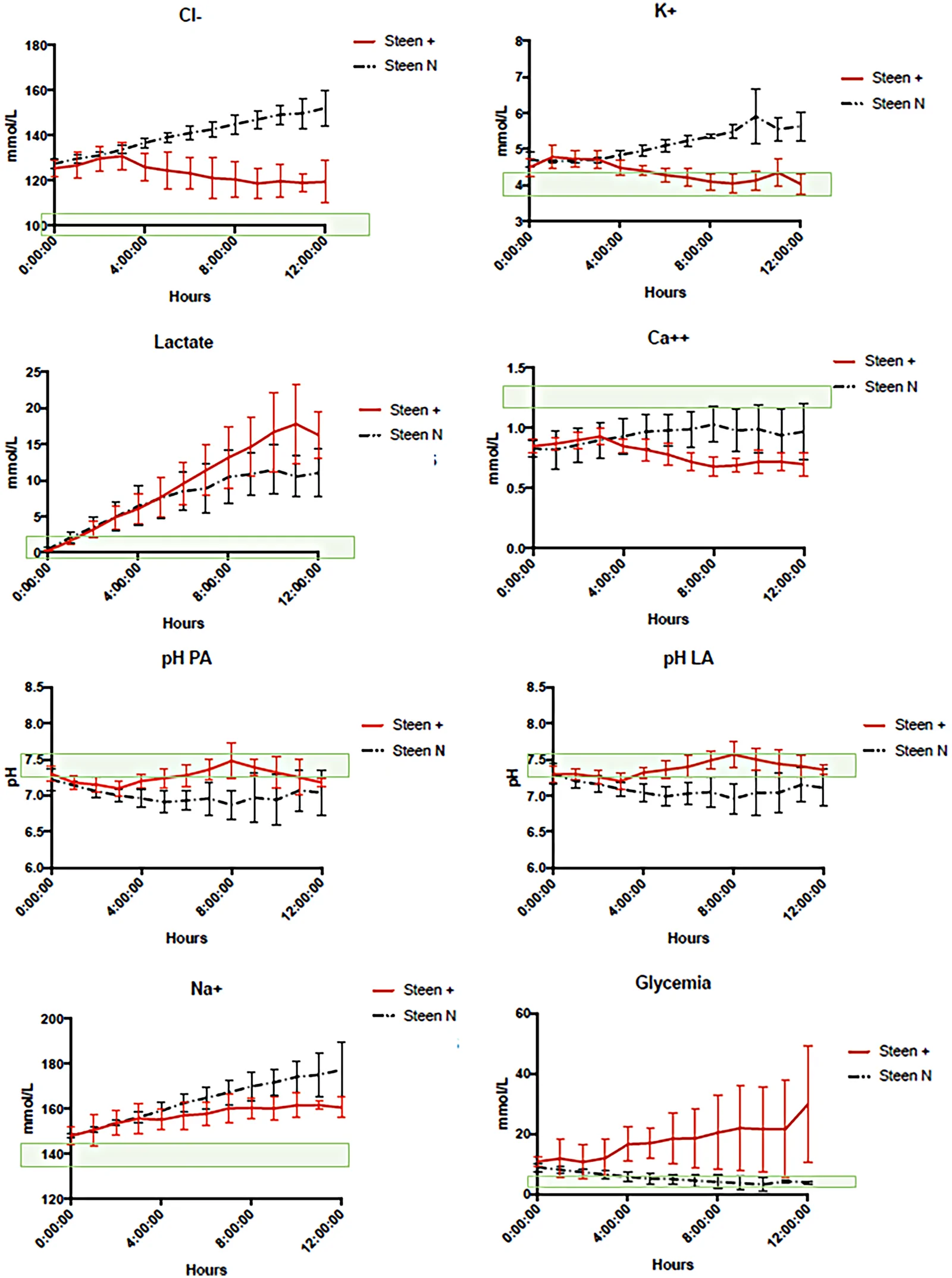
Evolution of electrolyte, lactate pH, Na+ and glucose levels in standard (steen N, *n*=5) and corrected (steen+*n*=5) perfusates along 12-hour EVLF. (Mean ± SD).

Pulmonary vascular resistance (PVR) did not change in both group (*p* = 0.55) neither over time (*p* = 0.49) nor group×time interaction (*p* = 1). PaO₂/FiO₂ ratios increased over time in both groups (time effect: *p* = 0.01) and was overall higher in the Steen + group (group effect: *p* < 0.001), with no significant group×time interaction (*p* = 0.69), indicating similar temporal patterns between groups (Figure 2). Compliance stayed significantly higher in the Steen + group (*p* < 0.001) whereas neither time (*p* = 0.46) nor interaction group×time (*p* = 1) reached statistical significance. For the perfusate pH: in the PA, 2-way repeated-measures ANOVA demonstrated a significant effect of group (*p* < 0.001) and a significant group×time interaction (*p* = 0.04), whereas the overall effect of time was not significant (*p* = 0.06). These findings indicate that differences between groups varied over the course of the study, despite the absence of a significant overall temporal trend. Similarly in the LA: 2-way repeated-measures ANOVA demonstrated a significant effect of group (*p* < 0.001) and a significant group×time interaction (*p* = 0.002), whereas the overall effect of time was not significant (*p* = 0.11). These findings indicate that differences between groups varied over the course of the study, despite the absence of a significant overall temporal trend (Figure 3). For lactate and electrolyte concentrations (Cl⁻, K⁺, Na⁺) 2-way repeated-measures ANOVA demonstrated in each measure a significant effect of group (*p* < 0.001 for each), time (*p* < 0.001 for each), and a significant group×time interaction (Lactate *p* = 0.01, Na⁺*p* = 0.01, Cl⁻: *p* < 0.001 and K⁺: *p* < 0.001). For glucose and calcium: 2-way repeated-measures ANOVA demonstrated a significant effect of group (*p* < 0.001) and a significant group×time interaction (*p* < 0.001), whereas the overall effect of time was not significant (Glucose: *p* = 0.9, Ca^2+^: *p* = 0.8).

### 3.3 Histology, electron microscopy, cytokines, elastometry, and protein expression

**Figure 4:**
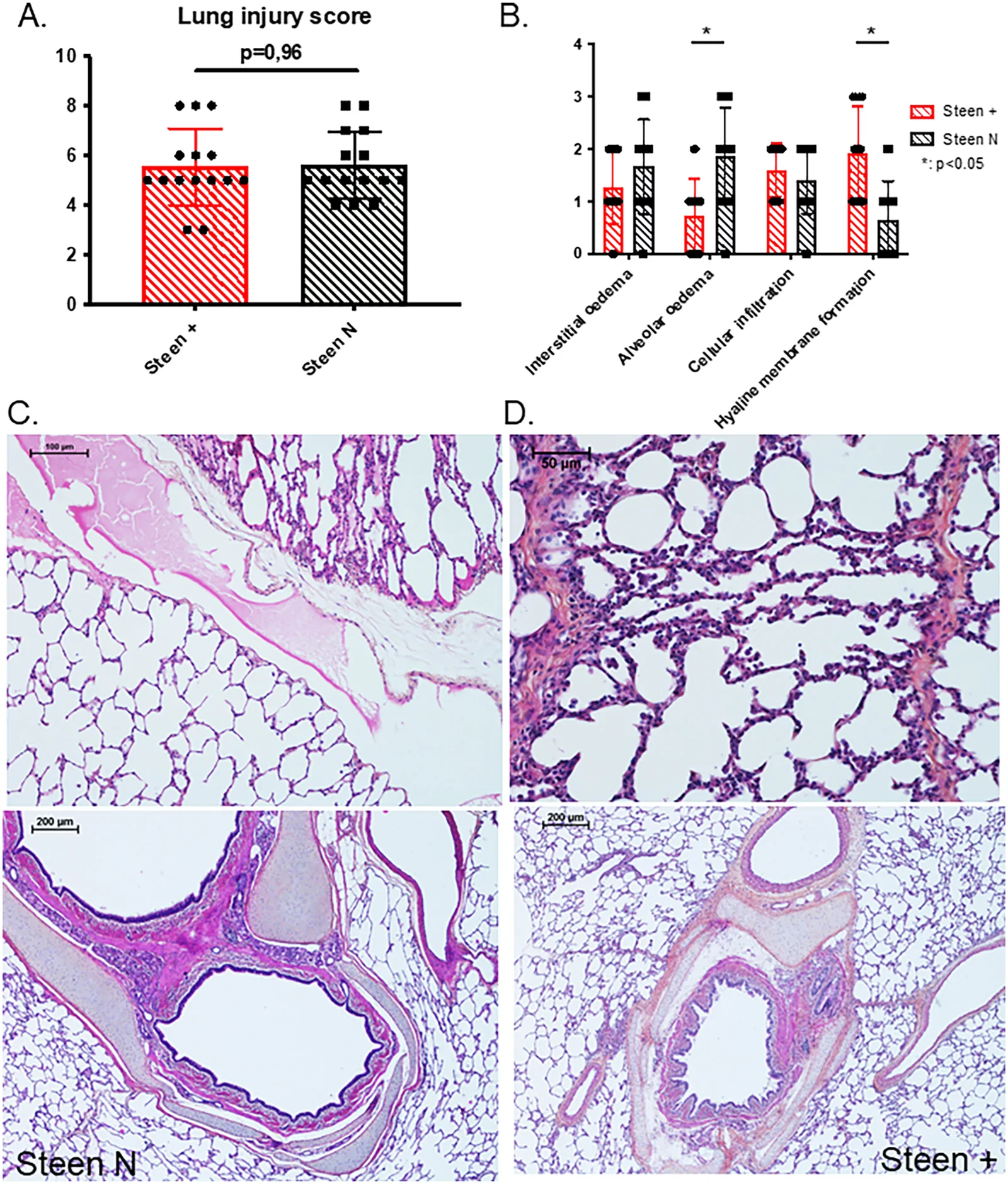
Pathology assessment of the right lung after 12 h of ex-vivo lung perfusion. **(A)** Total histological scores in the two groups (Steen N, *n*=5 and. Steen +, *n*=5), (Mean ± SD), (*t*-test comparaison); **(B)** Histological sub-scores in the two groups, (Mean ± SD), (*t*-test conaparaison); **(C)** Lung parenchyma after 12 h of ex-vivo lung perfusion in the standard. group (Steen N); **(D)** Lung parenchyma after 12 h of ex-vivo lung perfusion in the modified steer group (Steen +).

**Figure 5:**
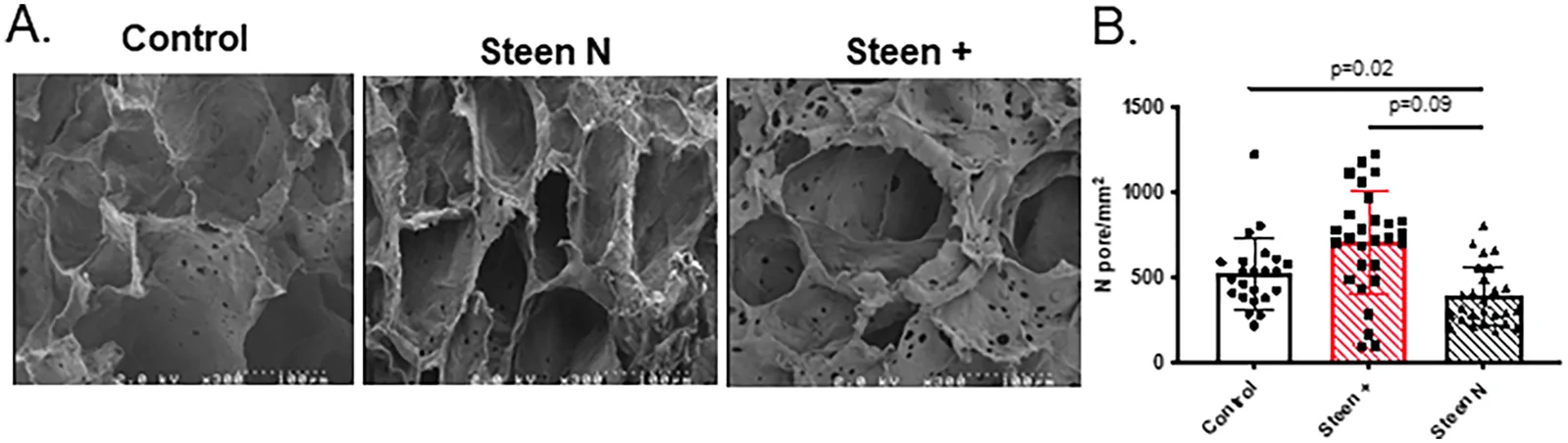
Electron microscopy assessment of the right lung after 12 h of ex-vivo lung perfusion. **(A)** Scanning electron microscopy of standard (Steen N, *n*=5) and corrected (Steen +, *n*=5) lungs after EVLP. The recipient pig left lung was used as a control. **(B)** Alveolar (Kohn) pore density (N/mm^2^) determined by scanning electron microscopy (Mean ± SD) (*t*-test comparaison).

**Figure 6:**
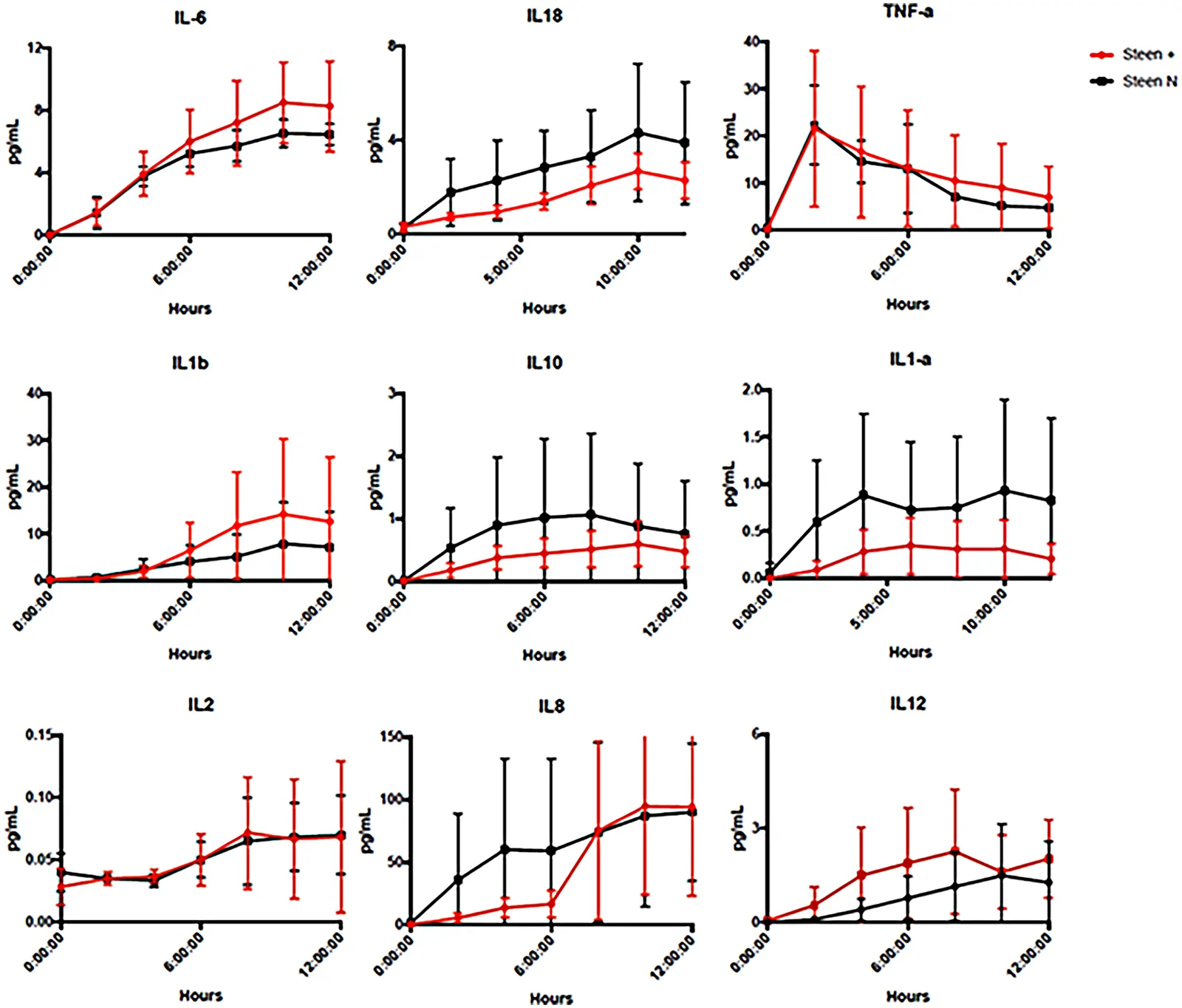
Evolution of cytokines levels in standard (steen N, *n*=5) and corrected (steen +. *n*=5) perfusates along 12-hour EVLP (Mean ± SD).

**Figure 7:**
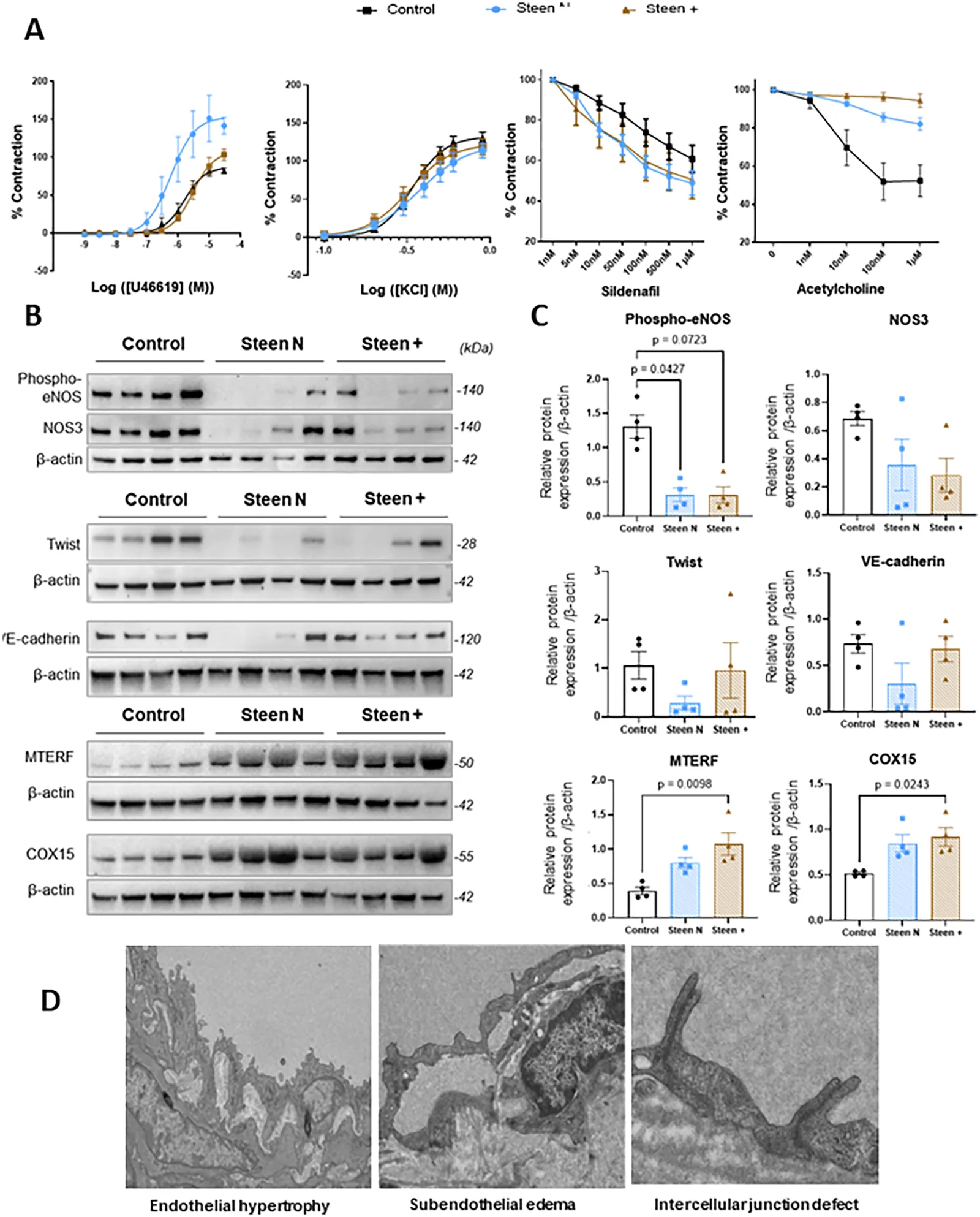
Consequences of 12-hour EVLP on endothelial and mitochondrial function. **(A)** Comparison of arterial contraction-relaxation capabilities in standard (Steen N, *n*=5) and corrected (Steen +, *n*=5) group after 12-hour EVLP. A non-perfused artery is used as control. U46619 (thromboxane A2 mimetic) dose-response curve, normalized to K90 (left) KCL dose-response curve, normalized to K90 (middle left); Sildenafil dose-response curve, reflecting smooth muscle cells-dependant relaxation after PA precontraction with U46619 (middle right); Acetylcholine dose-response curve, reflecting endothelium-dependant relaxation after PA precontraction with U46619 (right). **(B)** Western blots and **(C)** quantifications of endothelial and mitochondrial proteins expression in control and perfused. groups **(D)** Abnormalities observed in Steen+group by electron microscopy assessment of the right lung.

The semi-quantitative histological score was similar between groups (median 5 vs. 5; *P* = 0.96) (Figure 4). However, alveolar edema was lower in the corrected group (0.7 vs. 1.9; *P* < 0.001), while hyaline membrane formation was higher (1.9 vs. 0.7; *P* < 0.001). Transmission electron microscopy revealed endothelial hypertrophy, subendothelial edema, and intercellular junction abnormalities in the corrected group, whereas the standard group showed no such alterations (Figures 5, 7). Scanning electron microscopy demonstrated a significantly higher alveolar pore density in the corrected group compared with the standard group (711 ± 307 vs. 529 ± 210 pores/mm^3^; *P* = 0.02). Pore density did not differ significantly between the standard group and control lungs (529 ± 210 vs. 390 ± 173; *P* = 0.08). Perfusate cytokine levels (IL-6, IL-18, TNF-α, IL-1β, IL-10, IL-1*α*, IL-2, IL-8, IL-12) were analyzed over time, 2-way repeated-measures ANOVA demonstrated that no interactions were significant in any of the cytokine. For IL-6, IL-12 and IL-18 the group and the evolution over time differed significantly. For IL-1β, IL-2, IL-8 and TNF-α the level change over time, regardless of the treatment group (Steen N vs. Steen +). For IL-10 and IL-1*α* the groups differed overall, but their temporal evolution was similar (Figure 6). Pulmonary artery contractility and relaxation were assessed by myography. Maximal contraction with KCl was similar across groups. However, U46619-induced contractility was higher in the standard group compared with both corrected and control groups (Figure 7A), suggesting altered thromboxane-dependent contraction in the standard group. Sildenafil-induced relaxation (smooth muscle-dependent) was similar across groups, whereas acetylcholine-induced relaxation (endothelium-dependent) was significantly impaired in perfused lungs compared with controls, with greater impairment in the corrected group (Figure 7A). Western blot analysis revealed decreased NOS3 and phospho-eNOS expression in both perfused groups compared with controls, with further reductions in Twist and VE-cadherin, particularly in the standard group. Mitochondrial proteins MTERF and COX15 were upregulated in perfused lungs, significantly in the corrected group (Figures 7B,C). Electron microscopy confirmed endothelial structural alterations, including hypertrophy, subendothelial edema, and disrupted junctions (Figure 7D).

### 3.4 Outcomes after single-lung transplantation

**Figure 8:**
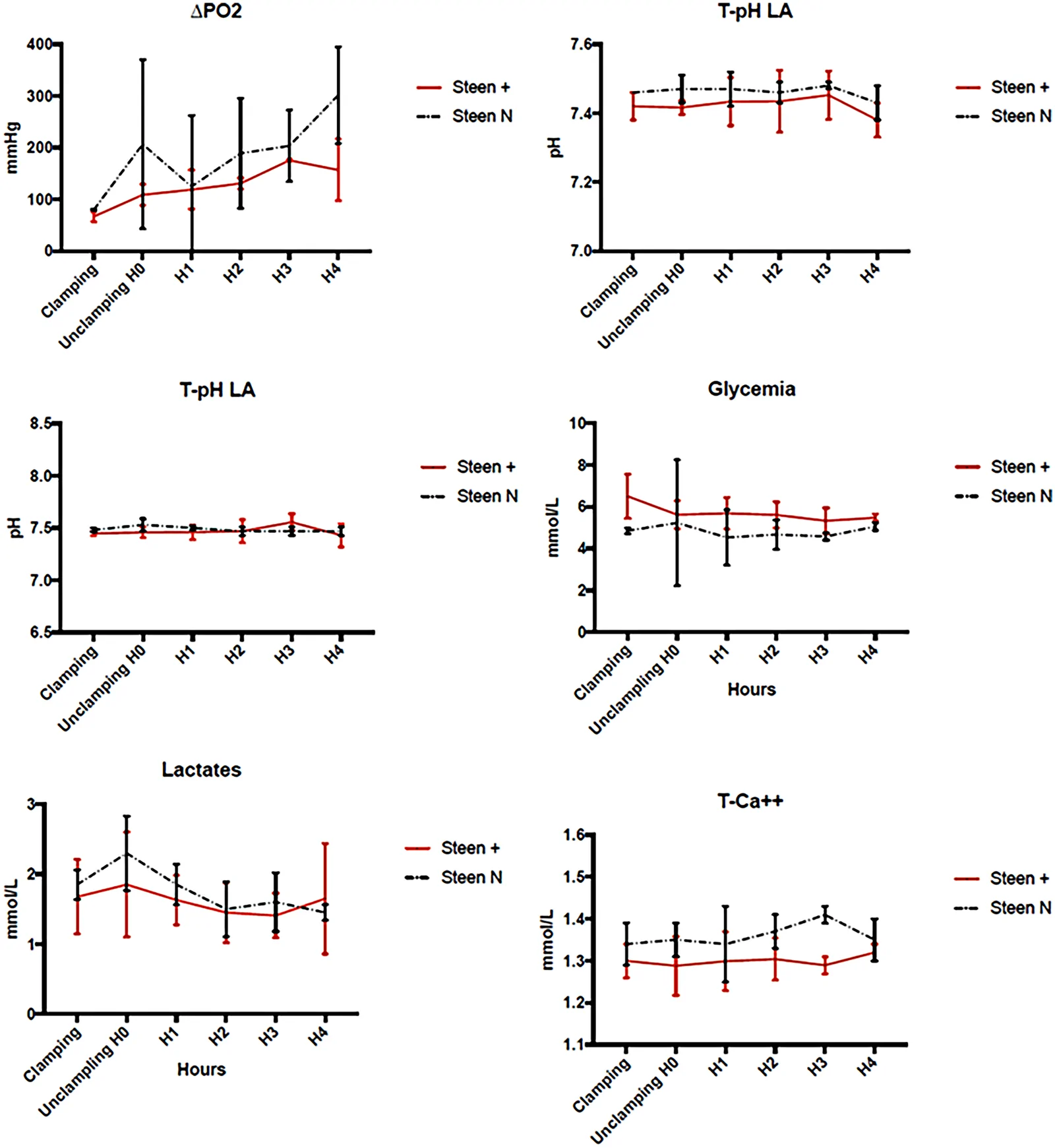
Evolution of *Δ*PO_2_, pH, Ca^2+^, glucose and lactate levels in standard (steen N, *n*=5) and corrected (steen +, *n*=5) groups after transplantation in recipient swines.

Post-transplantation, *Δ*PO₂ and PaO₂/FiO₂ ratios did not differ significantly between groups. Glucose, lactate, and pH values in PA and pulmonary vein samples were similar, as were electrolyte concentrations (Figure 8).

## 4 Discussion

In this study, we successfully maintained perfusate electrolyte and pH levels within physiological ranges throughout 12-hour EVLP in a porcine model. Perfusate correction appeared to play a meaningful role in stabilizing the EVLP environment, as electrolyte, pH, and glucose levels remained within their intended physiological ranges throughout perfusion. This more controlled biochemical milieu was associated with several indicators of improved lung quality, including enhanced compliance and oxygenation, reduced edema formation, and fewer hyaline membranes, along with a greater density of Kohn's pores after EVLP. Interestingly, despite these favorable trends, pulmonary vascular resistance did not differ between groups, and the absence of a significant post-transplant ΔPO₂ difference suggests that these structural and functional improvements during EVLP may not immediately translate into early post-implantation gas-exchange advantages. These findings highlight both the potential benefits and the current limitations of perfusate correction strategies. Our findings indicate that maintaining physiological perfusate composition during EVLP is feasible and may improve oxygenation and compliance without increasing PVR, supporting the safety of this approach. Glucose supplementation likely enhanced cellular metabolism, as suggested by higher lactate levels in the corrected group. However, hyperglycemia can lead to lung injury though oxidative stress and pulmonary vascular permeability, it also shapes the immune system facilitating infection (26, 27). Previous studies have shown that adding parenteral nutrition to the perfusate can prolong EVLP to 24 h and improve lung viability compared with glucose-only supplementation (28). Parenteral nutrition is associated with kidney and liver toxicity which is avoided in EVLP because the lung are isolated, unfortunately parenteral nutrition lead also to hyperglycemia (29–32). Thus, Steen solution, even with periodic replacement, may not provide sufficient metabolic support for extended EVLP. Adding glucose while avoiding hyperglycemia seems to be a valid option to investigate. Another factor of cell stability is a controlled pH (33, 34). In our study bicarbonate was added to successfully counteract pH decline associated with lactate accumulation. However it was associated with hypernatremia and we struggled to maintain a good sodium level during perfusion, other pH buffer may be investigated to maintain pH without ionic disorders (35). In clinical EVLP, elevated lactate or lactate/pyruvate ratios have not been linked to poor outcomes (36), however elevated lactate concentrations are not unequivocally indicative of enhanced aerobic metabolism and may equally reflect impaired lactate clearance, mitochondrial dysfunction, or cellular injury under metabolic stress. The higher lactate in the Steen + group can be either caused by enhanced glycolytic activity (supported by higher glucose availability) and/or the possibility of metabolic stress, particularly in light of the observed mitochondrial protein upregulation (MTERF, COX15). Oncotic pressure decreases during EVLP and may contribute to edema formation (14, 17, 37). Although Steen solution contains albumin to maintain oncotic pressure, we did not measure this parameter. The lower edema score in the corrected group suggests a potential benefit, but continuous monitoring or interventions such as hemofiltration may be necessary, as previously shown to reduce lung weight and improve compliance (14). Although correction of pH, electrolytes, and glucose allowed stable lung function over 12 h, our results suggest that Steen solution, even when periodically corrected, may not be sufficient as a standalone perfusate for truly prolonged EVLP. While pH and glucose homeostasis were achievable, important limitations persisted, particularly regarding edema control and endothelial preservation. Endothelium-dependent vasorelaxation remained significantly impaired, and ultrastructural analysis revealed persistent endothelial alterations, indicating that endothelial protection remains a critical unmet need. Perfusate cytokine levels typically rise during EVLP due to lung injury. In our study, cytokine concentrations were unaffected by correction. Adsorbent membrane filtration has been reported to reduce TNF-α and IL-8 levels without improving oxygenation or PVR (38), although other studies suggest additional benefits, including reduced edema and electrolyte imbalance (28). The higher hyaline membrane score and endothelial activation observed in the corrected group may reflect an inflammatory response to increased glucose availability. This could explain the impaired endothelial-dependent relaxation observed in elastometry testing. The pathological findings appear paradoxical, with reduced alveolar edema but increased hyaline membrane formation. In this context, hyaline membranes may reflect a reparative or inflammatory response to alveolar injury rather than ongoing edema. This process could be enhanced by greater glucose availability, which may promote a pro-inflammatory alveolar microenvironment. Alternatively, restoration of a more physiological metabolic milieu may have altered the protein composition of the perfusate, thereby favoring fibrin deposition and hyaline membrane formation. We hypothesize that macroscopic functional improvements and ultrastructural endothelial injury evolve on different timescales and are mediated through distinct biological pathways, which warrant further investigation. Greater alveolar pore density in the corrected group may enhance collateral ventilation and contribute to improved compliance and oxygenation (39–41). However, while increased alveolar pore density has been associated with enhanced collateral ventilation and may contribute to improved compliance, it has also been described in the context of emphysematous remodeling and structural lung injury. In our setting, the increase may reflect a combination of enhanced alveolar recruitment and tissue stress secondary to 12 h of normothermic perfusion, potentially exacerbated by the metabolic changes induced by glucose and bicarbonate supplementation. Finally, post-transplant outcomes were similar between groups, likely due to the presence of the native right lung, which limits interpretation of gas exchange data. Selective ventilation or bilateral transplantation would be required to fully assess graft function. The 4 h post-transplant follow-up and the confounding contribution of the native right lung are key limitations that preclude conclusions about long-term graft benefit. Another limitation is that the recipients were ventilated without selective intubation; therefore, post-transplant ventilatory indices reflect the combined contributions of the transplanted left lung and the native right lung, rather than the function of the graft alone. Selective right pulmonary artery clamping or bilateral transplantation would be required to isolate the contribution of the transplanted graft to post-operative gas exchange. This choice was dictated by ethical constraints and the survival design of the model The main limitation of our study is the relatively short EVLP duration (12 h), which may have been insufficient to reveal differences in long-term graft viability or reconditioning potential. Longer EVLP times could allow more extensive interventions, such as antibiotic therapy or gene transfer. Additional limitations include the fixed composition of the corrective solution and the lack of individualized adjustment. The small sample size constraint statistical power and limit the interpretation of the results. Nonetheless, minimizing animal use aligns with the 3Rs principle. These findings suggest that future protocols should move beyond simple biochemical correction and integrate targeted strategies aimed at maintaining endothelial integrity and capillary barrier function. Potential approaches include improved oncotic pressure management, modulation of glucose delivery to avoid hyperglycemic endothelial stress, and adjunctive techniques such as hemofiltration or adsorption to control metabolites and inflammatory mediators. In this context, deeper investigation of endothelial preservation mechanisms appears essential. Expertise in endothelial biology and vascular barrier regulation will be critical to refine perfusate composition and adjunctive interventions. Ongoing collaborative efforts within our group will specifically focus on endothelial metabolism, junctional stability, and mechano-transduction during prolonged EVLP, with the goal of limiting edema formation and preserving endothelium-dependent function The preservation of lung function observed in our study over 12 h supports the hypothesis that lungs can tolerate extended normothermic perfusion when physiological conditions are controlled. This strategy may support prolonged EVLP and facilitate lung reconditioning. Future studies should evaluate 24-hour EVLP protocols and explore adjunctive measures such as parenteral nutrition, complete normalization of biological parameters during EVLP, adsorbent membrane filtration, and devices replicating renal or hepatic function to optimize graft preservation. However, continuous EVLP may expose the endothelium to sustained metabolic and mechanical stress, contributing to endothelial dysfunction and edema (42). Intermittent EVLP, alternating perfusion and cold storage phases, may mitigate these effects by allowing metabolic recovery and limiting cumulative injury. At present, it remains unclear whether meaningful differences exist between 4-hour vs. 6-hour EVLP cycles using distinct perfusate strategies. Nevertheless, experimental and clinical data suggest that lungs preserved using intermittent EVLP maintain better functional stability over time. In our settings, we demonstrate an absence of early differences after transplant for lungs who underwent 12-hours corrected EVLP. In this context, it is conceivable that a strategy combining corrected perfusate composition with intermittent EVLP cycles could enable lung preservation for several days, potentially up to four days, while preserving functional and structural integrity (20). This hypothesis warrants dedicated experimental evaluation. Future studies should assess whether combining corrected perfusate composition with intermittent EVLP further improves endothelial preservation and limits edema formation during prolonged lung preservation. Optimization of glucose delivery within strict physiological ranges, alternative buffering strategies to stabilize pH without inducing hypernatremia, and active management of oncotic pressure represent key areas for refinement. Extending EVLP duration beyond 12 h, potentially over several days using intermittent strategies, may ultimately allow more effective lung reconditioning and expand graft availability for transplantation.

## Data Availability

The original contributions presented in the study are included in the article/Supplementary Material, further inquiries can be directed to the corresponding author.
